# Supplementary material for: Identification of the Tumor Infiltrating Lymphocytes (TILs) Landscape in Pure Squamous Cell Carcinoma of the Bladder
Source: Cancers (Basel). 2022 Aug 18;14(16):3999. doi: 10.3390/cancers14163999 (PMC9406640; doi:10.3390/cancers14163999)
Supplement: Supplementary file 1 [file cancers-14-03999-s001.zip › cancers-1824317-supplementary.pdf]

cancers MDPI

Supplementary Table S1. Patients' Characteristics.

**Parameter**		
**Age at RC**		
Median	66 years	
Range	37 – 86 years	
**Follow up after RC**		
Median	16 months	
Range	0 – 175 months	
	n	%
**Gender**		
Male	31	50.8
Female	30	49.2
**Tumor Stage**		
pT0	0	0
pT1	2	3.3
pT2	9	14.8
pT3	34	55.7
pT4	16	26.2
**Grading**		
G1	0	0
G2	25	41.0
G3	36	59.0
**Lymph Node Status**		
pN0	37	60.7
pN+	15	24.6
pNX	9	14.8
**Metastases**		
M0	56	91.8
M1	5	8.2
